# AGC1 Deficiency: Pathology and Molecular and Cellular Mechanisms of the Disease

**DOI:** 10.3390/ijms23010528

**Published:** 2022-01-04

**Authors:** Beatriz Pardo, Eduardo Herrada-Soler, Jorgina Satrústegui, Laura Contreras, Araceli del Arco

**Affiliations:** 1 Departamento de Biología Molecular, Universidad Autónoma de Madrid, 28049 Madrid, Spain; eherrada@cbm.csic.es (E.H.-S.); jsatrustegui@cbm.csic.es (J.S.); lcontreras@cbm.csic.es (L.C.); 2 Centro de Biología Molecular Severo Ochoa, Universidad Autónoma de Madrid (UAM)-Consejo Superior de Investigaciones Científicas (CSIC), 28049 Madrid, Spain; adelarco@cbm.csic.es; 3 Instituto de Investigaciones Sanitarias Fundación Jiménez Díaz (IIS-FJD), Universidad Autónoma de Madrid, 28049 Madrid, Spain; 4 Centro Regional de Investigaciones Biomédicas, Facultad de Ciencias Ambientales y Bioquímica, Universidad de Castilla La Mancha, 45071 Toledo, Spain

**Keywords:** malate-aspartate shuttle, AGC1/Aralar deficiency, mitochondrial disorders, mitochondrial aspartate-glutamate carrier, mitochondrial function

## Abstract

AGC1/Aralar/Slc25a12 is the mitochondrial carrier of aspartate-glutamate, the regulatory component of the NADH malate-aspartate shuttle (MAS) that transfers cytosolic redox power to neuronal mitochondria. The deficiency in AGC1/Aralar leads to the human rare disease named “early infantile epileptic encephalopathy 39” (EIEE 39, OMIM # 612949) characterized by epilepsy, hypotonia, arrested psychomotor neurodevelopment, hypo myelination and a drastic drop in brain aspartate (Asp) and *N*-acetylaspartate (NAA). Current evidence suggest that neurons are the main brain cell type expressing Aralar. However, paradoxically, glial functions such as myelin and Glutamine (Gln) synthesis are markedly impaired in AGC1 deficiency. Herein, we discuss the role of the AGC1/Aralar-MAS pathway in neuronal functions such as Asp and NAA synthesis, lactate use, respiration on glucose, glutamate (Glu) oxidation and other neurometabolic aspects. The possible mechanism triggering the pathophysiological findings in AGC1 deficiency, such as epilepsy and postnatal hypomyelination observed in humans and mice, are also included. Many of these mechanisms arise from findings in the *aralar*-KO mice model that extensively recapitulate the human disease including the astroglial failure to synthesize Gln and the dopamine (DA) mishandling in the nigrostriatal system. Epilepsy and DA mishandling are a direct consequence of the metabolic defect in neurons due to AGC1/Aralar deficiency. However, the deficits in myelin and Gln synthesis may be a consequence of neuronal affectation or a direct effect of AGC1/Aralar deficiency in glial cells. Further research is needed to clarify this question and delineate the transcellular metabolic fluxes that control brain functions. Finally, we discuss therapeutic approaches successfully used in AGC1-deficient patients and mice.

## 1. Introduction

**Figure 1:**
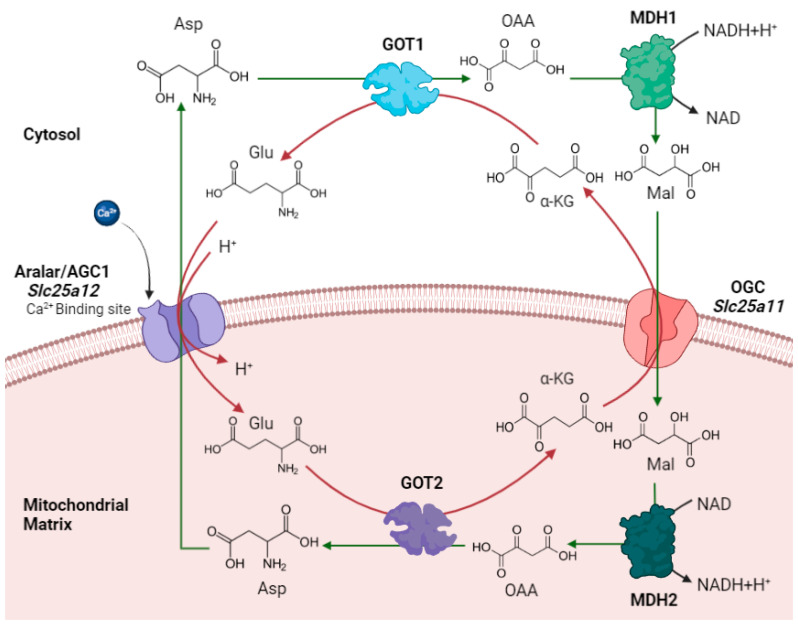
**The malate-aspartate NADH shuttle (MAS) as the major redox shuttle in brain.** The MAS is made up of four enzymes, mitochondrial and cytosolic aspartate aminotransferases (GOT2 and GOT1, respectively) and malate dehydrogenases (MDH2 and MDH1, respectively), and two mitochondrial carriers, located at the inner mitochondrial membrane, the α-ketoglutarate–malate carrier (OGC) and the aspartate-glutamate carrier (AGC). Note that AGC present a Ca^2+^ binding site that confers the Ca^2+^ sensitivity to MAS. Image created with BioRender.com.

The malate-aspartate shuttle (MAS) is considered to be the major NADH redox shuttle system in brain, mainly important in neurons. The function of MAS in brain is essential for maintaining a NAD^+^/NADH ratio favorable for the oxidative metabolism of glucose [1]. The MAS shuttle is composed by two pairs of cytosolic and mitochondrial enzymes (glutamate oxaloacetate transaminases (GOT) 1 and 2, and malate dehydrogenases (MDH) 1 and 2, respectively); and two mitochondrial carriers (the OGC/Slc25a11, oxoglutarate carrier; and the AGC/Slc25a12 or Slc25a13, aspartate-glutamate carrier; Figure 1).

Molecular cloning of AGC1/Slc25a12/aralar and AGC2/Slc25a13/citrin as the isoforms of the aspartate-glutamate carrier, the regulatory component of MAS [2,3], and the generation and characterization of *agc1*/*aralar*-KO mouse [4] opened up a new avenue to decipher the role of ARALAR-MAS pathway in brain metabolism. AGC1/Slc25a12/aralar and AGC2/Slc25a13/citrin have been found to be expressed by specific tissues; AGC1 mainly in excitable tissues as brain and muscle [5,6,7], and citrin in liver [8,9]. Consequently, a different phenotype is associated with the deficiency of each isoform in mouse models and human patients. Citrin deficiency causes a liver disease [8] whereas aralar deficiency provokes predominantly neurological effects [10]. Aralar-MAS deficiency in mice has been shown to produce severe neurological deficits with motor coordination defects and refractory epilepsy along with an impairment in myelination in the central nervous system [4]. The first human patient with AGC1/aralar deficiency presented with neurodevelopmental arrest, epilepsy and severe hypomyelination was described more than ten years ago [10]; and, more recently, other AGC1 deficient patients with early infantile epileptic encephalopathy were reported [11,12,13,14,15,16,17,18]. Most of these patients have homozygous or compound heterozygous mutations in *aralar* with low or no activity of the protein as a glutamate-aspartate carrier.

Since *aralar*-KO mice recapitulate the main hallmarks of the human disease, including developmental arrest, seizures, epileptic activity in hippocampus [19,20] and hypomyelination [4,21], the experimental work with these mice (lacking MAS activity in brain) has revealed new metabolic functions for ARALAR-MAS pathway. In the brain, *agc1*/*aralar* is mainly, if not exclusively, expressed in neurons [6,7,22,23], but its expression and functional relevance in astrocytes and oligodendrocytes is still controversial. In neurons, ARALAR was found to be particularly essential in the neuronal synthesis of Aspartate (Asp) and *N*-acetylaspartate (NAA) [4,24], and in the neuronal use of lactate as a fuel [25,26]. Moreover, basal respiration on glucose is reduced in *aralar*-KO neurons and the transmission of small Ca^2+^ signals to neuronal mitochondria is drastically impaired [25,27].

Herein, we will address the possible mechanism triggering the pathophysiological findings associated to AGC1/aralar deficiency, focusing on refractory epilepsy and postnatal hypomyelination observed in AGC1-deficient humans and mice. Other alterations related to AGC1 deficiency that have not been reported in humans but were identified in *aralar*-KO mice include: (1) the failure to synthesize Glutamine (Gln) in astrocytes [7] and (2) the dopamine (DA) mishandling in the nigrostriatal pathway [20]. Hypomyelination and deficits in Gln synthesis (Glutamate(Glu)-Gln cycle) may indicate that neuronal Aralar expression and a functional Aralar-MAS pathway is mandatory for the proper glial functions; although this point deserves further research. Other findings such as the refractory epilepsy and the susceptibility of the nigrostriatal DAergic system observed highlight the failure in neuronal energy metabolism associated with aralar-deficiency. Therapeutic approaches successfully used in patients [16,28] and mice AGC1-deficient (*aralar*-KO) [29] will be further discussed to better understand the metabolic transcellular fluxes in brain and the metabolic requirements in the different brain cell types affected by the lack of AGC1-MAS activity.

## 2. AGC1/Slc25a12/Aralar-MAS Pathway in Brain Metabolism

### 2.1. MAS as the Main NADH Shuttle in the Brain

An inability to maintain the balance of metabolic intermediates between the cytosol and mitochondria results in impairment of metabolism. In addition to many enzymes existing in either the cytoplasm or mitochondria, the pyridine nucleotides (NAD^+^, NADH, NADP^+^, and NADPH) are also compartmentalized between the cytoplasm and the mitochondria. Given that intact mitochondria are impermeable to NADH (but a mitochondrial NAD^+^ carrier has been described) [30], an indirect pathway involving the movement of electrons from NADH across the mitochondrial membranes must operate [1,31,32]. Thereby, the malate-aspartate shuttle (MAS), first described by Borst (1961) [33], is considered to be the major redox shuttle system transferring reducing equivalents from cytosolic NAD^+^/NADH to mitochondria that functions in brain. The MAS is essential for maintaining a NAD^+^/NADH ratio favorable for the oxidative metabolism of brain glucose.

The activity of the other major redox shuttle (glycerol-3-phosphate shuttle, GPS) in brain has been long questioned. Its activity in brain is strikingly low [34]. For a long time, the two enzymatic constituents of GPS (cytosolic and mitochondrial glycerol 3-phosphate dehydrogenases, cGPDH and mGPDH) were not found to co-localize in the same cell type, a fact that would be required for the GPS to be functional. This agrees with the absence of neurological dysfunction in GPS-deficient mice [35,36]. cGPDH was exclusively found in oligodendrocytes, where it could provide glycerol phosphate for phospholipid synthesis and mGPDH was found in neurons and astrocytes, where glycerol phosphate could be used as a respiratory substrate. However, transcriptomic studies in brain have revealed the coexistence of cGPDH and mGPDH in neurons and astrocytes [37]; and cGPDH and mGPDH are present in cultured astrocytes with similar activities [38], supporting the possible function of GPS in these cells. The role of GPS in other brain cell types is still unknown.

### 2.2. AGC1/ARALAR/SLC25a12, the Regulatory Component of MAS in Brain: Structure, and Cellular Expression

AGC catalyzes the electrogenic exchange of mitochondrial aspartate for cytosolic glutamate plus a H^+^ [3,39]. There are two AGCs in mammals, aralar/AGC1 [2] and citrin/AGC2 [8,9] encoded by genes SLC25A12 and SLC25A13 present on chromosomes 2q31 [9,40] and 7q21 [41], respectively. Aralar and citrin are both MAS components; and AGC2/citrin, the liver isoform, is also a part of the urea cycle [3,42]. AGC1/aralar/Slc25a12 was found to be mainly expressed in excitable tissues such as brain, muscle and heart; and AGC2/citrin/ Slc25a13 was found in tissues such as liver [5]. Mutations in both isoforms have been associated with human pathologies, those of AGC1 to the early infantile epileptic encephalopathy 39 (EIEE39) with the first clinical case reported by Wibom et al. (2009) [10]; and those of citrin to idiopathic neonatal hepatitis (NICCD), failure to thrive and dyslipidemia caused by Citrin deficiency (FTTDCD) and adult-onset type II citrullinaemia (CTLN2) [8,43,44,45].

The AGCs belong to a distinct subfamily of mitochondrial carriers, the Ca^2+^-binding mitochondrial carriers (CaMCs) [24] that have N-terminal extensions harboring EF-hand Ca^2+^-binding motifs and are regulated by cytosolic Ca^2+^ [2,3,25,46,47]. These isoforms, AGC1/Aralar/Slc25a12 and AGC2/citrin/Slc25a13, provide stimulation by Ca^2+^ of MAS activity within the 150–300 nM range; slightly higher for aralar than for citrin [25,48].

Aralar and Citrin have a unique three-domain-structure, consisting of an N-terminal domain with eight EF-hand Ca^2+^-binding motifs (of about 300 amino acids) only one of which really binds Ca^2+^, a carrier domain involved in transport (of about 300 amino acids), and a C-terminus with an α-helix domain [49]. The carrier domain consists of six transmembrane α-helices sharing structural determinants with members of mitochondrial carrier family. Most of the structural information for these transporters was achieved from studies with the ADP/ATP carrier bound to specific inhibitors [50,51]. This approach enabled structural analysis in specific conformations; with the substrate-binding site accessible to the intermembrane space or to the matrix, the cytoplasmic (c-) and matrix (m-) state, respectively, providing relevant insights on the mechanisms involved in the translocation of substrates (for details see Ruprecht and Kunji, 2020, 2021; and references therein) [52,53]. Briefly, two salt-bridge networks established between conserved residues alternatively open and close the carrier to one or other side of the membrane allowing the access of substrates to an internal substrate-binding site. Charged residues of highly conserved motifs located in the odd- and even-numbered transmembrane α-helices participate, respectively, in the formation of these matrix and cytoplasmic salt-bridges [52]. Additionally, a single substrate-binding site formed by conserved residues from the even-numbered α-helices was identified in the middle of the central cavity. Thus, the substrates initially bind to the c- or m-state and trigger conformational changes that alter these salt-bridge networks facilitating the translocation of the substrates across the carrier [54]. Because of the high homology displayed by members of mitochondrial carrier family, the transition between states might constitute a general mechanism. Consequently, in AGCs such as in other mitochondrial carriers, mutations in these residues may produce defective transport of their substrates through the inner mitochondrial membrane and be associated with the origin of several human diseases as EIEE39 or Citrullinaemia type II.

Furthermore, the structure of Ca^2+^-bound and Ca^2+^-free forms for the N-terminal regulatory domain of the human AGCs has been solved providing relevant data regarding the mechanism of activation by Ca^2+^ [55]. This domain has two functional modules, a mobile unit formed by EF-hands 1–3 responsible of the regulation by Ca^2+^ and the other consisting of EF-hands 4–8, which have lost the Ca^2+^-binding function and shapes a rigid dimerization interface making the carrier a structural dimer [55]. When Ca^2+^ binds to EF-hand 2, the only one with Ca^2+^-binding function, this module is displaced, increasing the accessibility of the substrates to the transport domain and, in turn, the transport activity of AGCs. Similarly, mutations in the relevant residues of this regulatory domain may originate non-functional disease-associated alleles [55].

As mentioned above, AGC1/ARALAR protein is mainly found in excitable tissues such as brain [2,5], where it is the major isoform in neurons [6,7,22,23]. Surprisingly, in mouse brain, AGC2/CITRIN is also present in neurons, although only in restricted brain areas and at low levels [56]. However, other reports claim the existence of aralar mRNA expression [57,58] and protein expression in astrocytes from mouse brain [58]. Regarding the expression of *aralar* in oligodendrocytes, there is no evidence of the protein ARALAR in mouse oligodendrocytes in vivo [7], and transcriptomic analysis revealed that oligodendrocyte precursor cells (OPCs) and mature oligodendrocytes do express aralar mRNA but at lower levels than neurons [37,59]. As well, several works report that AGC1/aralar deficiency produces changes in OPC proliferation and maturation [21,60,61]. These controversial data about *Aralar* expression raise the importance of further deciphering the role of Aralar-MAS in specific brain cell types.

### 2.3. Use of Experimental Models of AGC1 Deficiency Revealed New Functions of ARALAR-MAS

#### 2.3.1. Experimental Models of Mice with AGC1 Deficiency (Aralar-KO Mice)

Two *aralar*-KO mice have been generated so far, and they differ in the disruption sites and mouse strains used, intron 13 and hybrid SVJ129 × C57BL/6 by Jalil et al. (2005) [4] and exon 1 and pure C57BL/6 by Sakurai et al. (2010) [60]. However, both have similar phenotypes, indicating that the effects of the disruption are not compensated by the influence of modifying genes polymorphic between the two strains. *Aralar*-KO mice were growth-retarded, exhibited generalized tremor, seizures and had pronounced motor coordination defects along with an impaired myelination in the central nervous system [4,60]. Biochemical analysis in brain and cortical neuronal cell cultures revealed drastic decreases in Asp and NAA levels as the main biochemical markers for AGC1/aralar deficiency [4]; as well as severe reductions in alanine and serine in all brain regions [20]. Most of the *aralar*-KO mice die at about 20–22 days of age regardless of the genetic background, and few of these animals in the hybrid SVJ129 × C57BL/6 strain occasionally reach 27–30 days [4]. We often witnessed spontaneous or audiogenic motor tonic-clonic seizure from which the *aralar*-KO mice did not recover; however, we cannot ascertain whether seizures contributed to animal death (discussed in Section 4.1) [19].

Although *aralar* is mainly expressed in neurons, no neuronal cell death has been reported in brains from *aralar*-KO mice [4,20,21,26]. However, at 20 postnatal day (PND), *aralar*-KO brain has a pronounced loss of neurofilament (NF)-containing processes in striatum and cerebral cortex layers, a loss that Sakurai et al. (2010) [60] coincided with an altered cell alignment in the Purkinje cell layer at 13–14 PND. Interestingly, NF loss is not general for all neurons in the *aralar*-KO brain, insofar as no loss of NFs in cerebellar Purkinje neurons was observed. A pronounced loss of myelinated fibers was found in all of these gray matter regions in the *aralar*-KO mouse, clearly showing that the loss of neuronal processes is independent of the myelination defect. The postnatal hypomyelination has been associated with a fall in NAA levels in *aralar*-KO neurons that could result in a limited synthesis of galactocerebrosides, a major component of myelin lipids in oligodendrocytes (discussed in Section 4.2) [4,21,24].

In *aralar*-KO mice in vivo, anaplerotic synthesis of astroglial Glu and Gln was found to be highly reduced [7]. This points to a gradual impairment of glutamatergic transmission in these animals, which is likely to contribute to the severity of the phenotype (discussed in the following Section 4.3). Moreover, *aralar*-KO mice have a lack of motor coordination in the hindlimbs with no “gross” muscle effects. They also show hyperactivity, anxiety-like behavior, and hyperreactivity with a decrease of dopamine in terminal-rich region as the striatum. These results indicate that the nigrostriatal dopaminergic system constitutes a target of AGC1/Aralar deficiency (discussed in Section 4.4) [20].

#### 2.3.2. New Aralar Functions Discovered in Aralar-KO Mice

In this section, we will discuss the involvement of the Aralar-MAS pathway in neuronal functions such as glucose and lactate metabolism and Asp formation; as well as the role that Aralar-MAS plays in the Glu–Gln cycle.

As mentioned above, although *aralar* is mainly expressed in neurons, no neuronal cell death was detected in brains from *aralar*-KO mice [4,20,21,26]. However, neuronal glucose metabolism is severely compromised in cultured *aralar*-KO corticohippocampal neurons using glucose as main fuel. Basal respiration is reduced in *aralar*-KO neurons and the upregulation of respiration in response to neuronal activation is specially compromised [27]. Neurons respond to different stimuli (veratridine, high K^+^-depolarization, Glu, NMDA) which mobilize Ca^2+^ and increase neuronal workload by stimulating respiration. This is required to rebuild the ATP broken down to restore the resting state. Remarkably, *aralar-*KO neurons fail to stimulate respiration to the same degree as control neurons [26,27,29,62,63,64]. Stimulation of respiration is accompanied by increases in mitochondrial NAD(P)H, as determined by two-photon microscopy imaging and these increases are blunted in the absence of ARALAR [25], supporting the notion that a Ca^2+^-dependent increase in MAS activity is involved. In addition, the supply of exogenous pyruvate fully reverts the decreased response to stimulation in terms of mitochondrial NAD(P)H levels and respiration of *aralar*-KO neurons, showing that by activating MAS, cytosolic NADH is reoxidized shifting pyruvate away from LDH and lactate production and favoring its entry and oxidation in mitochondria.

As a member of the major redox shuttle in neurons, the absence of ARALAR leads to an inability to metabolize lactate [25,26] and thus severely compromises the function of the astrocyte-to-neuron lactate shuttle, which has an important protective role under neurodegenerative and physiological conditions [65,66,67]. This aspect will be further discussed in Section 4.1.

Brain Asp synthesis. Results from *aralar-*KO mice showed that brain Asp levels depend on the presence of ARALAR. Indeed, Asp levels in the brains of *aralar-*KO mice drop about 80–90%, [4,24]. Asp is the precursor of NAA, which is depleted in the *aralar*-KO brain, and this will be treated separately in the context of myelination (see Section 4.2). Asp is the amino acid with highest levels in mitochondria [68] where it is formed from OAA and Glu thanks to GOT2 activity. Asp levels are much higher in neurons than astrocytes [69], in agreement with their higher levels of ARALAR [7]. *Aralar* is required to take up Glu from the cytosol in exchange of Asp provided by the matrix. Consequently, the lack of Aralar explains the fall in whole brain Asp. In agreement with the requirement of cytosolic Glu for matrix Asp formation in neurons, a smaller albeit consistent decrease in brain Asp levels has been detected in brains from mice with a neuron-restricted deletion in the Glu transporter GLT-1/EAAT2/Slc1a2 [70,71,72].

At this point, a word of caution is appropriate. Ralphe et al. (2004) [73] and other groups [74] described the presence of the plasma membrane Glu-Asp carriers (EAATs) in mitochondria and attributed to EAATs a role as mitochondrial Asp/Glu carriers. Although we showed that the activity of AGC1 and AGC2 accounts for all MAS activity in heart [75], it is still possible that EAATs in certain cell types may be mitochondrial proteins. However, the fact that removal of a plasma membrane Glu transporter EAAT2 in neurons causes an unbalance of brain Asp similar to that caused by Aralar deletion as noted above [70,71] may explain the findings in T cells in which EAAT1/Slc1a3 knockdown caused metabolite changes interpreted as due to removal of a bona fide AGC [76].

Brain Gln synthesis. As ARALAR is mainly present in neurons it came as a surprise that the in vivo synthesis of Gln, a classical glial-born amino acid, was defective in the *aralar*-KO brain [7]. Astrocytes from *aralar*-KO mice do not show any gross impairment in glucose utilization [38] or in the respiratory response to various workloads when using glucose as fuel [77,78]. The clue to this paradox lies in the pathway utilized by astrocytes to build the carbon backbone of Glu from glucose thanks to the exclusive presence of pyruvate carboxylase (PC) in these cells [72,79]. PC converts some of the pyruvate arising from glucose into OAA, the main anaplerotic metabolite in the TCA, which, after reaction with acetylCoA (also arising from pyruvate), is converted into citrate. In the TCA, citrate is converted into α-KG, in which all carbons arise from glucose.

The possible role of neuronal Aralar lies at the level of the formation of Glu from α-KG. In the case of cerebral hyperammonemia, excess ammonia is disposed of in the form of Gln exported from the brain. This requires de novo formation of Glu in astrocytes and it has been shown that astrocytic Glu dehydrogenase (GDH) plays an essential role in the reductive amination of α-KG to Glu using ammonia [80]. However, in normoammonemia, GDH is mainly involved in Glu deamination [81] and the preferred route of Glu formation from α-KG is transamination in the cytosol with GOT1 as major contributor as shown from the preference for Asp versus other amino acids as amino donor for astrocyte Glu formation [7,72,82]. We have argued that Asp arising from neurons is an amino donor for astrocyte transamination of α-KG, and that the low levels of Asp observed in *aralar*-KO neurons explain the lack of astrocyte Glu and Gln synthesis in *aralar*-KO brain [7]. Moreover, a similar symbiotic relationship for glial Glu + Gln formation between photoreceptors (which provide Asp) and Muller glial cells has also been described [83,84]. However, it remains to be determined whether the very low AGC1 levels in astrocytes are also able to provide some cytosolic Asp for Glu synthesis in these cells as proposed by Hertz and Rothman [82]. In any case, the fall in Gln and Glu in *aralar-*KO brain points to a gradual impairment of glutamatergic transmission in these animals, which is likely to contribute to the severity of the phenotype (discussed in the Section 4.3).

Glu oxidation in brain mitochondria. Current evidence indicates a limited role of AGC1/Aralar in brain Glu oxidation. Indeed, GDH appears to be the major route for Glu oxidation in mitochondria not only in astrocytes and synaptosomes [81,85,86] but also in neurons [87].

**Figure 2:**
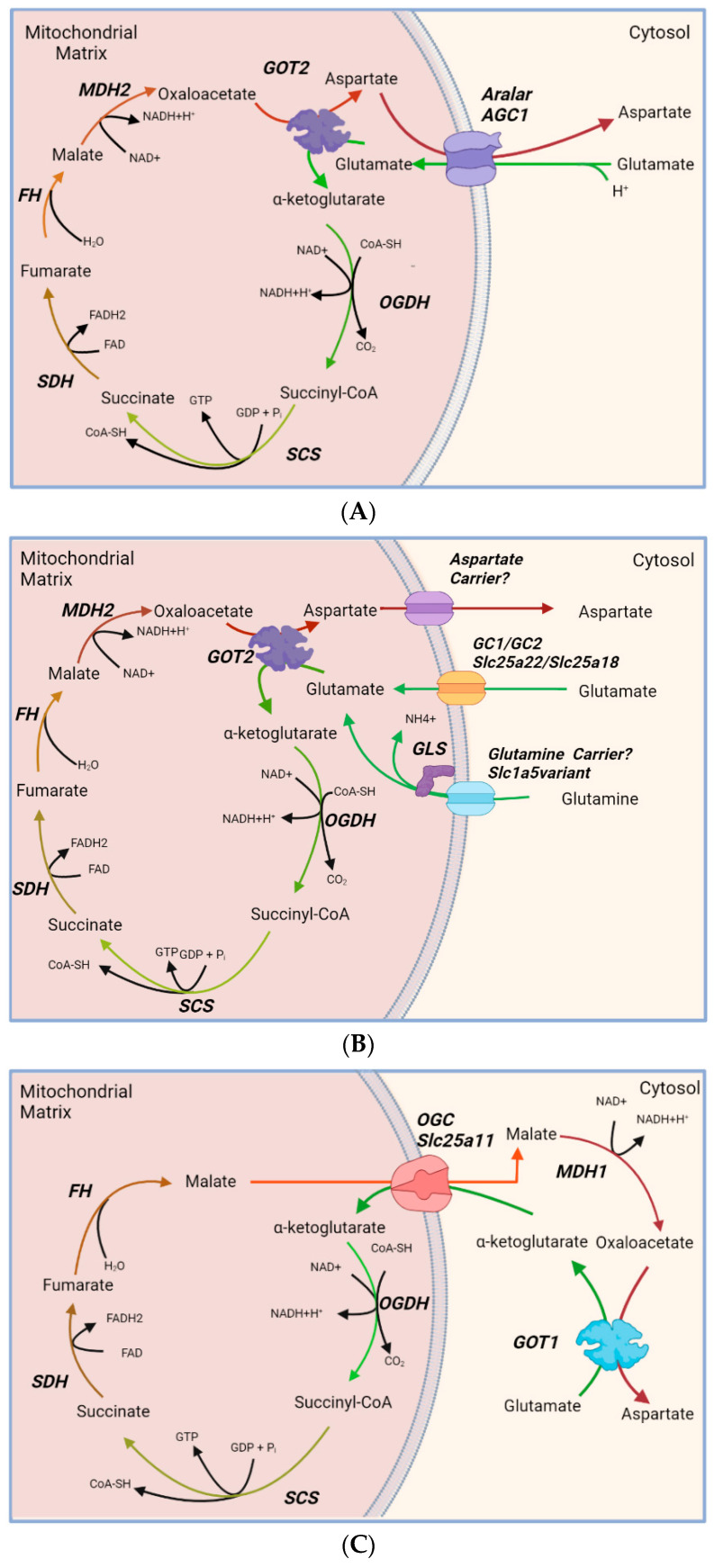
**Glutamate (Glu) oxidation in brain mitochondria and truncated TCA cycle**. Glu is transported inside the mitochondria through AGC1/Aralar, where it is oxidized in the truncated TCA cycle and converted to aspartate (Asp) by the mitochondrial GOT2. Asp exits the mitochondria using AGC1/Aralar (**A**). In the absence of AGC1, formation of aspartate from Glu may occur as follows: (1) Glu is either transported inside the mitochondria via the GC1/GC2, or synthesized from glutamine (Gln) which enters the mitochondria through a Gln Carrier. After the oxidation cycle, Asp exit from the mitochondrial matrix is mediated by a non-AGC1 Asp carrier (**B**); (2) Glu oxidation takes place in mitochondria but the transamination leading to Asp formation takes place in the cytosol via cytosolic GOT1. α-ketoglutarate enters the mitochondria in exchange of malate via the reversible OGC to feed the truncated TCA cycle. (**C**) In this scenario one of the oxidation steps of truncated TCA occurs in the cytosol via MDH1. This may be favored under glucose deprivation as NADH levels drop substantially [91]. AGC, Asp-Glu carrier 1; FH, fumarate hydratase; GC1/GC2, Glu carrier 1 or 2; GLS, Glutaminase; GOT1 and GOT2, glutamic-oxaloacetic transaminase 1 and 2; MDH1 and MDH2, malate dehydrogenase 1 and 2; OGC, oxoglutarate-malate carrier; OGDH, oxoglutarate dehydrogenase; SCS, succinyl-CoA synthetase; SDH, succinate dehydrogenase. Image created with BioRender.com.

However, AGC1/Aralar could participate in a possible truncated TCA cycle [81,86,88], in which mitochondria is energized thanks to Glu oxidation in mitochondria up to OAA, leaving out the reactions in which acetylCoA is incorporated from citrate synthase to isocitrate dehydrogenase. This truncated TCA cycle is proposed to function typically under glucose deprivation conditions [89] and results in the accumulation of Asp at the expense of Glu, an abundant amino acid in neurons. A similar situation arises in astrocytes in which GDH, the major Glu oxidative pathway, is deleted [86] and in retina upon inhibition of the mitochondrial pyruvate carrier by zaprinast [90]. The formation of Asp is thought to be mitochondrial, via GOT2, and would require Asp exit from mitochondria via Aralar (Figure 2A). Thus, it is surprising that a truncated TCA cycle with increases in the Asp/Glu ratio was also detected in zaprinast treated *aralar*-KO retinas [90]. Furthermore, a truncated TCA cycle has been shown to function upon disruption of GDH, the major Glu oxidation pathway in astrocytes [86] which have very low Aralar levels. Therefore, if Aralar is not a component of the truncated TCA cycle in neurons, it is possible that other mitochondrial Asp carriers exist (Figure 2B), or that the transaminase reaction occurs in the cytosol via GOT1, with α-KG entry in mitochondria in exchange of malate via the readily reversible OGC (Figure 2C). These two steps, GOT1 and OGC are also part of MAS, but working in reverse. It should be borne in mind that the truncated TCA cycle functions when there is no glucose, and therefore a limited driving force (i.e., NADH/NAD^+^ ratio) for MAS function.

## 3. AGC1/Aralar- and MAS-Deficiency in Humans

Several AGC1/Aralar mutations have been reported to produce AGC1 deficiency in humans [10,11,12,13,14,15,16,17,18]. AGC1 deficiency, named “early infantile epileptic encephalopathy 39” (EIEE39; OMIM ID # 612949; DEE39, “Developmental and Epileptic Encephalopathy 39”), is an inborn error of metabolism affecting AGC1-MAS activity and producing symptoms that include neurodevelopmental delay, hypomyelination, refractory epilepsy, and severe hypotonia.

### 3.1. Mutations in AGC1 Related to Human AGC1 Deficiency

EIEE39 is an autosomal recessive disease caused mainly by homozygous mutation in the *SLC25A12* gene (see Table 1). The first case of human AGC1-deficiency/EIEE39 was reported by Wibom et al. (2009) [10]. The patient, a 3-year-old girl, presented episodes of apnea and sporadic tonic seizures with focal clonic jerks. Physical examination revealed severe muscular hypotonia, and psychomotor retardation. In this patient, brain magnetic resonance imaging (MRI) scans showed enlarged ventricles, prominent cortical sulci, with lack of myelination in cerebral hemispheres but normal myelination in cerebellum and brainstem. Interestingly, the globus pallidus and putamen showed some loss of volumen but no focal lesions were found in the cortical gray matter. Magnetic resonance spectroscopy (MRS) showed severely reduced NAA levels, although normal choline and lactate signals with increased myoinositol. Molecular analysis revealed a homozygous missense mutation at position 590 (c.1769A > G; p.Gln590Arg) in the AGC1 protein. This mutant form of AGC1 was unable to transport aspartate or glutamate [10]. Gln590 is a highly conserved residue that protrudes into the internal cavity of the transporter just above the proposed substrate-binding site [92]. Therefore, it seems likely that its substitution by Arg results in the trapping of the substrate at the binding site of the carrier, hindering its movement through the protein.

Later, Falk et al. (2014) [11] described two related cases of AGC1 deficiency. Similarly to the former patient, these two siblings presented profound developmental delay, muscular hypotonia, refractory epilepsy, abnormal myelination, cerebral atrophy and fluctuating basal ganglia changes consistent with suspected metabolic disease. Brain MRS showed decreased NAA, but increased choline, myoinosytol and lactate (in CSF and parenchyma). The affected individuals presented a homozygous missense mutation (c.1058G > A; p.Arg353Gln). The affected residue, Arg353, resides in the first transmembrane α-helix (TM1) domain and is found highly conserved not only between AGC isoforms and Glu carriers but also in many other MCs, thereby confirming its relevance in the function and/or structure of these transporters. Moreover, this residue is located just below the matrix gate and it is thought to participate in closing and opening the carrier on the matrix side through the interaction with Glu384, a highly conserved residue of TM2 [93]. Indeed, AGC1 activity was found reduced to 15% of wild type in the p.Arg353Gln variant [11].

Subsequently, whole-exome sequencing (WES) analysis of undiagnosed pediatric patients allowed for the identification of more cases of AGC1 deficiency, such as those reported by Pronicka et al. (2016) [13], Nashabat et al. (2019) [15] and Kose et al. (2021) [18] showing different homozygous missense pathogenic variants in *SLC25A12* (c.1335C > A, p.Asn445Lys; c.1385C > T, p.Thr462Met; and, c.125G > C, p.Arg42Pro, respectively), presenting epileptic encephalopathy and global developmental delay. Surprisingly, a recent case reported by Pfeiffer et al. (2020) [16] describing a homozygous missense variant in the same region (c.1331C > T; p.Thr444Ile) suffered refractory seizures and developmental arrest but did not have evidence of cerebral hypomyelination on MRI demonstrated at 10 months of age. This patient also had decreased NAA, elevated myoinositol and choline peaks with no lactate peak evidenced on MRS. Although no hypomyelination was evidenced at the age tested; it is plausible that the myelination defects might be later in onset for this proband. The altered residues reside in the TM3 α-helix, and these substitutions are considered likely to impact secondary protein structure and that are predicted to be damaging. More recently, Saleh et al. (2020) [17] described also a case of cerebral palsy with epilepsy and severe global developmental delay. Brain MRI showed diffuse brain atrophy, with decreased cortical white matter and enlarged ventricles. By WES analysis a homozygous nonsense mutation (c.400C > T; p.Arg134Stop was identified. This variant would lead to a premature stop codon and truncated protein or mRNA degradation by the nonsense-mediated decay pathway.

In the case reported by Parnes et al. (2015) [12] a compound heterozygous mutation in *SLC25A12* produced psychomotor regression with dystonia, indicative of extrapyramidal effects, and spasticity. Interestingly, levodopa treatment led to a modest improvement of muscular tone consistent with a movement disorder. WES analysis revealed two previously undescribed mutations, a frameshift mutation in exon 4 (c.2956C > T/p.K100fs) and a missense variant (c.215T > C; p.I72T) affecting the calcium-binding regulatory domain, both predicted to be damaging. Kavanaugh et al. (2019) [14] have also reported one individual compound heterozygous for *AGC1* mutations (c.1295C > T; p.A432V and c.1447-2_1447-1delAG). The p.A432V variant also involves a residue from the TM3 α-helix and appears to alter the secondary structure and packing of the protein. The c.1447-2_1447-1delAG deletion removes a splice acceptor site which may result in the loss of exon 15 and subsequent frameshift leading to the truncation of the transport domain (this variant will produce a complete loss-of-function). This patient presented with epilepsy, hypomyelination, hypotonia, severe intellectual disability and spastic quadriplegia (spasticity as previously described by Parnes et al. 2015 [12]). They diagnosed their patient based on MRI results as suffering leukodystrophy of the leuko-axonopathy category (mechanisms stemming from neuronal and/or axonal defects), as proposed by Van der Knaap and Bugiani (2017) [94], rather than a primary hypomyelinating disorder.

Intriguingly, in AGC1-deficient humans, unlike other mitochondrial diseases which affect neurons and glia, brain lactate accumulation is only observed in some patients related to seizures. By using the *aralar*-KO mouse, we also found that ARALAR deficiency does not produce a primary increase in brain lactate [38]. Brain Lactate is largely produced by astrocytes which have very low ARALAR levels, and therefore do not modify lactate production in AGC1 deficiency [38]. AGC1/Aralar functions primarily in neurons in which upregulation of glycolysis is limited [95,96] explaining the lack of brain lactate accumulation in AGC1 deficiency.

### 3.2. Other Inborn MAS Deficiencies in Humans

Deficiencies in the enzymatic components of MAS, namely GOT2, MDH1 or MDH2 enzymes, have been recently reported as infantile-onset encephalopathies with epilepsy, abnormalities in myelination, hypoplasia and brain atrophy [97,98,99]. Similar symptoms to those of AGC1 deficiency (see Section 3.1). This clinical phenotype is consistent with the fact that the MAS is particularly important for the CNS [100]. Accordingly, MDHs, GOTs, and OGC, like AGC1 [2,5], show high expression in excitable tissues such as brain, heart, and skeletal muscle [101].

In the inborn MAS disease caused by defective GOT2, four affected children from independent families have been reported to date [99]. Three of them presenting homozygous missense mutations and the fourth proband with a compound heterozygous mutation (in-frame deletion and missense variant). All variants affect conserved amino acids that may reduce the activity of the enzyme GOT2. Interestingly, these bi-allelic GOT2 mutations produced a lack of serine synthesis and a defect in one-carbon metabolism. This observation emphasizes that the MAS pathway is required for the synthesis of metabolites such as serine that require NAD^+^ in the cytosol. The epilepsy and the overall neurodevelopmental status in these patients were pyridoxine and serine responsive [99]. Remarkably, a drastic drop in serine together with Asp was also detected in brain from *agc1*-KO mice [7,20]. In GOT2 deficiency, mitochondrial Asp production is decreased; and this lack of Asp will lead to hypercitrullinaemia and dysfunction of the urea cycle. In the inborn error of metabolism (IEM) caused by defective MDH1, the deleterious variant is a missense homozygous mutation affecting the conserved NAD^+^-binding domain of the protein [98]. The patients are two consanguineous cousins with a severe neurodevelopmental disease presenting elevated glicerol-3P (G3P) concentrations in blood but no change in lactate. The high levels of blood G3P, a potential biomarker in MDH1 deficiency, might reflect increased G3P shuttle function as a compensatory mechanism for generating cytosolic NAD^+^. MDH2 deficiency has been reported in three unrelated patients presenting bi-allelic missense pathogenic mutations [97]. These patients suffer generalized hypotonia, psychomotor delay, refractory epilepsy and have elevated lactate in the blood and cerebrospinal fluid. No clear biomarkers were identified in these patients, since MDH2 is more ubiquitously expressed than other MAS genes, probably reflecting its direct involvement in the TCA cycle.

## 4. AGC1 Deficiency: Pathophysiology

In this section, we will address the possible mechanisms of the pathophysiology of AGC1 deficiency, focusing on refractory epilepsy and postnatal hypomyelination observed in both AGC1-deficient humans and mice as well as the failure to synthesize Gln in astrocytes [7], and DA mishandling in the nigrostriatal pathway [20] seen in *aralar*-KO mice.

### 4.1. Epilepsy

AGC1 deficiency is an epileptic encephalopathy that is refractory to anticonvulsants in humans. All patients experienced their first seizures within a few months after birth, as well as apneic episodes, focal and generalized seizures [10,11,14,16]. Some of these patients were fed a ketogenic diet (KD) with a drastic positive effect on seizure control [16,28]. Interestingly, epilepsy is frequently encountered in patients with IEMs. In fact, IEMs as those linked to impaired glucose or pyruvate oxidation, such as mutations in glucose transporter 1 (GLUT-1), with reduced brain GLUT1 (G1D syndrome), mutations in pyruvate dehydrogenase (PDH) or in the mitochondrial pyruvate carrier (MPC deficiency) are currently associated with developmental delay, abnormalities of muscle tone, cerebral hypomyelination and hyperexcitability with refractory epileptogenic activity [102,103,104]. All symptoms also reported for the human pathology associated to AGC1 deficiency.

Broadly, epilepsies associated with IEM frequently display age-dependent presentation that presumably is related to the sequential development of excitatory and inhibitory pathways in the neonatal brain. GABA functions initially as an excitatory neurotransmitter (NT) in the premature infant and a developmental switch changes its role to inhibition closer to term. This switch occurs as a result of the maturation of the cation-chloride cotransporter (KCC2). Subsequently, there is a surge of glutamatergic-related excitatory connections, which favors excitability during this developmental phase. The immaturity of inhibitory systems during early brain development and their dysregulation under metabolic dysfunction play a major role in neonates by lowering the seizure threshold and favoring epileptogenesis. Many IEMs are associated with failure in important functions of brain metabolism such as the transport and utilization of energy substrates, the production of energy-rich phosphates, and the metabolic coupling between neurons and astrocytes. Disturbances in the NT pathways with excess of excitation or lack of inhibition in the immature brain can enhance seizure activity. However, other mechanisms such as substrate deficiency (e.g., serine deficiency), or malformations of cortical development may be also associated with epilepsy (e.g., PDH deficiency; for review, Sharma et al. 2017 [103])

Paradoxically, a large number of neuropathologies in which neurons experience a deficit in oxidative phosphorylation and in ATP levels are frequently accompanied by severe seizures. This is the case for AGC1 deficiency or G1D syndrome that are associated with hyperexcitability rather than hypoexcitability [4,20,29,102,105]. This fact is paradoxical and intriguing because neuronal overexcitation always imposes a high demand of ATP in neurons that are critically lacking metabolic resources. So, the question is posed as how neurons can be hyperexcitable with reduced levels of ATP. Related to this, it has been recently shown that neurons with a deficient MPC are intrinsically hyperexcitable as a consequence of impaired calcium homeostasis, which reduces M-type potassium channel activity [104]. Interestingly, loss of function of these M-current generating KCNQ channels (KCNQ2 and KCNQ3) causes epilepsy in humans and mice (for review, De la Rossa et al. 2020 [104]).

In AGC1 deficiency, several factors might contribute to the etiology of epilepsy. First, AGC1/Aralar deficiency decreases energy supply. *Aralar*-KO neurons display half-capacity mitochondrial respiration in physiological glucose concentrations [27]. Pyruvate formation is also decreased and pyruvate supplementation can restore mitochondrial respiration in *aralar*-KO neurons. Second, unbalanced neurotransmission results from deficits in astroglial Glut/Gln synthesis as observed in brain from *aralar*-KO mice [7]. Third, Impaired brain development and brain atrophy have been found in AGC1-deficient humans and mice. As observed in the AGC1-deficient humans, *aralar*-KO mice also suffer life-long seizures (tonic-clonic motor seizures) until death at PND20–22. In control mice, seizures disappear from PND11 when the excitatory-inhibitory circuit reaches maturity [19,20]. In the *aralar*-KO brain, the abnormal synaptic and spike electrogenesis in the CA3/hilus region evolved to status epilepticus. This aberrant neuronal electrogenesis may result from disturbed substrate supply to neuronal mitochondria [19]. Notably, the high responsiveness of AGC1-deficient humans to KD [16,28] emphasizes that the epileptic phenotype displayed in the AGC1-deficient patients seems to be mainly metabolic in origin and is unlikely to be the consequence of neuronal network remodeling.

Additionally, a dysfunctional MAS caused by AGC1/Aralar failure produces a block in lactate utilization by neurons [25,85], which prevents the protective role of lactate on neuronal excitotoxicity. This fact was demonstrated both in *aralar*-KO neurons in vitro and in Kainate-treated *aralar*^+/−^ mice in vivo [26]. Lactate now stands as an alternative energy source during excitotoxic brain injury, since neurons recover more effectively from excitotoxicity when using lactate rather than glucose alone [105]. During epilepsy, brain Glu and lactate increase. Lack of Aralar-MAS activity limits Glu-induced stimulation of neuronal mitochondrial respiration; and enhances cytosolic ATP/ADP ratio drop upon Glu exposure. In fact, *aralar*^+/−^ mice are more susceptible to excitotoxicity probably because a failure to use lactate is detrimental [26]. This hypersensitizing effect to excitotoxicity could also occur in Aralar-MAS deficient patients.

### 4.2. Postnatal Hypomyelination

Brain from both AGC1/Aralar-deficient humans and mice display hypomyelination and decreased NAA levels in vivo [4,10,11,13,14,15,17,29,60], with the exception of the human case reported by Pfeiffer et al. (2020) [16]. In AGC1-deficient mice, a clear decrease in the major myelin-specific lipid galactocerebroside and myelin-specific proteins was found in the brain with no differences in the lipids from peripheral nerves such as sciatic [4]. The data suggest that the failure in postnatal myelin synthesis occurs exclusively in the central but not in the peripheral nervous system of AGC1-deficient probands. However, curiously, the hypomyelination in the patients seems to be confined to the cerebral hemispheres, with findings essentially normal in the cerebellum and brainstem [10]. Perhaps, since the ontogeny of tissue-specific expression of AGC1 in the human CNS is unknown, residual expression of AGC2 may explain the regional differences seen in patients [10,56].

Hypomyelination in AGC1 deficiency has been attributed to neuronal failure in NAA production. NAA is the second most abundant metabolite after Glu in the human CNS [106], and emits the largest signal in ^1^H-NMR spectroscopy of the human and mouse brain. Hypomyelination would be caused by a lack of neuronal Asp and consequently NAA. Transaxonal transport of NAA through the NAA transporter NaDC3 and cleavage by oligodendroglial aspartoacylase (ASPA), would be required to supply acetyl groups to developing oligodendrocytes for myelin lipid synthesis [4,21,24], energy metabolism or other functions. Other groups [14] support that MRI findings in AGC1 deficient patients are most consistent with a leukodystrophy of the leuko-axonopathy category, (i.e., mechanisms stemming from neuronal and/or axonal defects), as previously proposed by Van der Knaap and Bugiani (2017) [94], and not a primary hypomyelinating disorder.

However, it is surprising that oligodendrocytes would use NAA rather than glucose for their own lipid synthesis, at least during development. This may be related to the distribution of hypomyelination in *aralar*-KO mice, which is more pronounced in gray than in white matter regions [21]. The growing process in a myelinating oligodendrocyte is farthest apart from its own cell body, but it lies in close contact with the axon around which it grows and wraps [107,108]. It is possible that local lipid synthesis occurs at the growing tip of the oligodendrocyte, using NAA from the axon that it wraps as precursor [21]. However, it is unclear that healthy oligodendrocytes would use NAA rather than glucose or other substrates as ketone bodies (KB), for their own lipid synthesis during postnatal myelination. In fact, in *aralar-*KO subjects KBs supply resumed myelination bypassing the metabolic restriction imposed by aralar-MAS deficiency, in both humans and mice [28,29].

These results indicate that the hypothesis that hypomyelination is a consequence of the lack of neuronal NAA production needs further investigation. Although, NAA is mainly localized in neurons [109,110] it cannot be ruled out that immature oligodendrocytes are able to produce NAA [69,111] and perhaps Asp. Canavan disease (CD) is a severe leukodystrophy caused by ASPA deficiency leading to accumulation of its substrate, NAA, and spongy myelin degeneration. Pathogenic mechanisms in CD are still unknown and might involve both the lack of NAA-derived acetate as precursor for myelin lipids and the osmotic effect of NAA accumulation. Recently, Maier et al. (2015) [112] and Guo et al. (2015) [113] reported that NAA-derived acetate is not essential for myelin lipid synthesis and its loss does not cause the myelin phenotype in CD mouse model with ASPA deficiency. In addition, Sohn et al. (2017) [114] showed that neuronal loss in these mice is suppressed by preventing NAA synthesis. In addition, a patient with defective Asp-NAT gene lacking NAA synthase activity and brain NAA had only a slightly delayed myelination as revealed by MRI [115]. These results cast doubt on the role of neuronal NAA as an obligatory precursor for fatty acid synthesis in oligodendrocytes.

During postnatal myelination, axonal ensheathment is completed within 12–18 h and during this time oligodendrocytes elaborate about 3 times their weight in membrane per day. At that time oligodendrocytes must have extremely high metabolic rates, and consume large amounts of oxygen and ATP. In fact, mitochondrial function seems to be necessary for immature but not for mature oligodendrocytes which undergo a metabolic switch and survive in vivo by aerobic glycolysis [116]. In relation to the effects of AGC1/Aralar deficiency on oligodendrocytes, several reports describe changes in maturation and OPC proliferation. Ramos et al. (2011) [21] reported that *aralar*-KO brain display an increase in immature oligodendrocytes. In addition, Sakurai et al. (2010) [60] found that pure rat oligodendrocyte cultures in which *aralar* has been silenced have a maturation defect. In addition, AGC1 deficiency was found to induce a deficit in OPC proliferation in vitro with precocious differentiation into oligodendrocytes; and OPC reduction in *aralar^+/−^* mice [61]. Clearly, the possible impact of AGC1 deficiency on oligodendrocyte-dependent postnatal myelination and in the demyelination–remyelination processes associated with in human diseases as multiple sclerosis awaits further study.

### 4.3. Failure in Astroglial Glu and Gln Synthesis

It is well-known that Glu is the main excitatory neurotransmitter in the CNS. The neuronal release of Glu is followed by uptake into astrocytes rather than neurons. This continuous drain of Glu from neurons to astrocytes is compensated through the supply of Gln, a Glu precursor formed in astrocytes. Thereby, glutamatergic neurons rely on an active Glu–Gln cycle with neighboring astrocytes to maintain glutamatergic neurotransmission [117]. However, in addition to transcellular cycling, about 10% to 30% of the Glu–Gln is oxidized under basal conditions [118,119] leading to a net loss of these compounds from the cycle. The lack of pyruvate carboxylase in neurons makes them incapable of de novo synthesis of Glu and also GABA from glucose [120]. This requires a continuous replenishment of Glu and Gln in astrocytes. Therefore, the small glial pool of Glu, precursor of the Gln, is rapidly turning over. De novo Glu and Gln production in astrocytes requires the supply of one or two ammonia groups, respectively, and neurons are thought to supply one or both (reviewed in Bak et al. 2006) [117]. Interestingly, studies on the *aralar-*KO mouse have indicated that neuronal-born Asp is the nitrogen donor required for de novo Glu production in the neighboring astrocytes, leading to a gradual failure of the Glu–Gln cycle in these animals (Pardo et al. 2011 [7]; see Section 2.3.2).

Curiously, the activity of the Glu–Gln cycle may be compromised in certain forms of epilepsy [121,122] that have been associated with reactive astrogliosis. Reactive astrocytes, other than cellular hypertrophy and enhanced expression of GFAP, also manifest a reduced expression of Gln synthetase in some types of epilepsy. This finding has led to the hypothesis that Glu–Gln cycle deficits associated with astrogliosis may be relevant to the pathogenesis of epilepsy [121,123,124]. These observations need to be kept in mind to evaluate the posible link between the Glu–Gln cycle deficit [7] and the epilepsy observed in the AGC1 deficient probands, in which astrogliosis has been previously reported [21].

### 4.4. Deficits in the Nigrostriatal DAergic System

At the behavioral level, *aralar*-KO mice show deficits in motor coordination, tremor, ataxic gait, seizures, hyperreactivity, anxiety-like behavior, demotivation and hyperactivity [4,20]. All these observations raised the question whether damage to specific neuronal cell groups in the brain, preferentially affected by AGC1/Aralar deficiency, drives the phenotype of *aralar*-KO mice; and therefore that observed in patients.

In fact, the striatum was found to be specifically affected by AGC1/Aralar deficiency. DAergic neurons in the striatum, but not in the limbic system, showed decreased DA and vesicular monoamine transporter 2 (VMAT2), and increased DA catabolism and oxidative stress [20]. Further studies on healthy adult mice expressing only half-a-dose of *aralar* also revealed that these mice display an increased metabolism of DA by monoamine oxidase (enhanced DOPAC/DA ratio) and an enhanced sensitivity to amphetamine. These findings support that the striatum is a preferential target of Aralar-MAS deficiency which leads to a reduction and/or mishandling of DA and that oxidative stress caused by AGC1/Aralar deficiency might be at the origin of DA mishandling in striatum. Interestingly, fluctuating changes in the basal ganglia by MRI were reported in AGC1-deficient patients associated with motor disturbances, spasticity [10,11,18] and dystonia with a discrete response to levodopa treatment [12].

The failure of the nigrostriatal pathway in AGC1/Aralar deficiency can be reasonably explained since Aralar-MAS supplies NADH to complex I of the electron transport chain. So, it can produce a failure in the complex I activity due to a lack of substrate, to which certain neuronal populations, such as the DAergic of the nigrostriatal pathway, will be more susceptible [125,126,127]. Thus, the inhibited Aralar-MAS pathway in Aralar deficiency and in OXPHOS defects might further contribute to neurodegeneration in Parkinson disease (PD).

## 5. Treatment of Patients and Murine Models of AGC1/Aralar Deficiency

### 5.1. Treatment of AGC1-Deficient Patients with Ketogenic Diet (KD)

Falk et al. [11] proposed KD as a possible therapy for AGC1 deficiency in 2014, but it was not until 2015 that the effect of this diet was reported in an AGC1 deficient patient [28]. The first patient described for AGC1/Aralar deficiency [10] initiated a treatment with KD at the age of 6 years and for at least 19 months. At this time, the response of this patient to the treatment was reported to be dramatic [28], presenting no more seizures and a clear improvement in psychomotor development and resumed myelination. Sometime later, Pfeiffer et al. (2019) [16] also tried the benefits of KD in the treatment of this rare neurometabolic disorder. Their patient started a KD, with significant improvement in control of his seizures. Seizure frequency abated from a few per week to none in 4 months since starting a KD.

KD has a high fat content (80–90%) with little but sufficient protein, and a drastic reduction in carbohydrates that leads to a switch from glucose to ketogenic metabolism. KD contains both long-chain and medium-chain fatty acids, which give rise to KBs in the liver, increasing the KB: glucose ratio in circulation. In both KD-treated patients with AGC1 deficiency [16,28] the diet was composed of fat to nonfat in a ratio of 4:1. The rationale for resumed myelination with this diet is based firstly on that KD bypasses the metabolic block in AGC1 deficiency by providing KBs as an alternative fuel to glucose for the brain; and, secondly, that by reducing glycolytically generated NADH, cytosolic MDH1 enzyme shifts the equilibrium towards OAA formation, resulting in cytosolic Asp production and compensating for the abolished mitochondrial efflux of Asp [28]. On the other hand, KBs from KD may confer resistance against epileptic seizures by several proposed mechanisms, namely, (1) their effect on ionic channels, (2) inducing changes in gene expression that involve BDNF expression, (3) a direct inhibitory effects of the KB βOHB on histone deacetylase, and, (4) by leading to changes in the balance of excitatory versus Inhibitory NTs (for review, Katsu-Jiménez et al. 2017) [128]. In fact, KD has also proven beneficial in several other metabolic diseases associated with pharmaco-resistant epilepsy and hypomyelination [129,130,131,132,133,134]. In brief, KD improves the symptomatology associated with AGC1 deficiency in humans [16,28]. The future will tell us how far the patients’ improvement will proceed, and how much damage is irreversible. In light of the beneficial treatment of these AGC1-deficient cases, identification of additional affected patients at a younger age has become extremely important.

### 5.2. Treatment of Agc1/Aralar- KO Mice with KD or with the KB, β-Hydroxybutyrate (βOHB)

As previously described, KD has been successfully used in patients with AGC1/Aralar deficiency, restoring myelination [28] and preventing epilepsy [16,28], two of the main hallmarks of this rare disease [10]. However, although the benefits of long-term KD were significant, the clinical recovery was moderate in humans perhaps because the onset of the treatment was at an advanced neurodevelopmental stage [28]. To assess the therapeutic potential of KD, the diet was administered earlier to *aralar*^+/−^ mice females from pregnancy or during the postnatal life of the *aralar*-deficient pups, but unfortunately, testing KD on mice was unfeasible since it affected fertility and induced mice mortality [29]. To solve this question, βOHB, the main KB produced during KD, with anticonvulsant and neuroprotective properties as KD [135,136,137] was also tested in the KO mouse. Recent data by Pérez-Liébana et al. (2020) [29] revealed important recovery effects of βOHB administration on brain function of *aralar*-KO mice under glucose unrestricted conditions.

A brief treatment of *aralar*-KO pups with βOHB elicited a marked positive effect on Asp and NAA production, postnatal myelination, and DA homeostasis [29]. Curiously, short term treatment with βOHB also recovers DAergic neurons from neurotoxicity induced by inhibition of mitochondrial complex I activity [138,139,140]. *Aralar*-KO mitochondria have no defects in complex I but rather a depletion of the main respiratory substrate, pyruvate, and low NADH levels [25,27]. In *aralar*-KO brain, recovery of striatal DAergic neurons is most likely due to mitochondrial consumption of βOHB enhancing mitochondrial NADH production, respiration and ATP synthesis [29], as shown in PD mice models [139]. Additionally, βOHB-induced recovery of mitochondrial NADH may prevent mitochondrial ROS production and loss of cytosolic VMAT2, affected by AGC1 deficiency, which will allow for recovery of normal DA homeostasis in the nigrostriatal terminals [20,141]. In addition, βOHB restored deficits in both basal and Glu-stimulated mitochondrial respiration of aralar-deficient neuronal cultures. Thus, βOHB constitutes an effective substrate able to bypass the energetic limitation imposed by AGC1/Aralar deficiency in neurons.

Impaired myelin synthesis in AGC1/Aralar deficiency has been attributed to a lack of neuron-born NAA used as precursor for postnatal myelin lipid synthesis [4,10,21,24,28]. βOHB oxidation in mitochondria boosted the neuronal synthesis of cytosolic Asp and NAA, impeded by aralar deficiency [29], presumably through the citrate-malate shuttle. Cytosolic citrate may lead to oxaloacetate (through ATP citrate lyase); and given the low Asp and α-Ketoglutarate levels in the cytosol of *aralar*-KO neurons, Asp aminotransferase may favor cytosolic Asp synthesis. This pathway is ARALAR independent and would allow neuronal NAA formation available for transaxonal transport into oligodendrocytes for myelin lipid synthesis [7,21,24]. However, myelin recovery obtained after intraperitoneal βOHB was not associated with a measurable increase in Asp nor NAA in the brain of *aralar*-KO mice [29]. Similarly, no increase in brain NAA was reported in the AGC1/Aralar-deficient patient with increased myelination resulting from KD [28]. The fact that neither Asp nor NAA levels were increased in the brains of βOHB-treated *aralar*-KO mice is probably because of their continuous use possibly by nearby glial cells. Obviously, it seems reasonable to think that oligodendrocytes can also directly use βOHB as a precursor for myelin lipid synthesis. However, the contribution of βOHB-derived vs NAA-derived acetyl CoA to the overall postnatal myelination process in oligodendrocytes remains unclear.

These results indicate that βOHB supplementation, the main metabolic product of KD, under conditions of no carbohydrate-restriction might be adequate for improving AGC1 deficiency. In any case, the high levels of plasmatic βOHB entails a reduced glucose utilization in peripheral tissues by the known “Randle effect” [142]. Understanding the specific role of βOHB in the effects of KD has a special interest in AGC1 deficiency because only KB, but not KD lipids, are metabolized by neurons [143]. Since the AGC1/Aralar deficiency pathology further entails a restriction to the neuronal use of glucose; and, the high-lipid low-carbohydrate KD is unpalatable and present difficulties for long-term adherence and undesirable health consequences, the usefulness of βOHB for human therapy should be evaluated in future.

## 6. Conclusions

AGC1/Aralar is the Asp-Glu mitochondrial carrier and the regulatory component of the NADH MAS activity, mainly expressed in excitable tissue such as neuronal brain cells. AGC1-deficiency in humans, mostly due to mutant ARALAR with defective transport activity, provokes the “early infantile encephalopathy 39” (EIEE39) inducing a neurodevelopmental arrest similar to that observed in the *aralar*-KO mice, which represent a good model for studying the human disease. In brain, neurons highly express *aralar* and depend on the ARALAR-MAS pathway for energy metabolism with glucose as substrate. Neuronal functions such as the Asp and NAA synthesis, lactate consumption, respiration and the response to small Ca^2+^ signals mediating the Ca^2+^-induced activation of the mitochondrial metabolism are dependent on ARALAR-MAS activity.

*Aralar* expression and the relevance of Aralar-MAS function is limited in glial cells but, paradoxically, some of the most important glial functions (as astroglial Gln synthesis and postnatal myelination by oligodendrocytes) are drastically affected by AGC1/Aralar deficiency. Herein, we discuss the glial affectation based on a secondary aralar-dependent neuronal damage as the most possible mechanism involved. The lack of neuronal metabolic supply of Asp to glia may mediate the shortage in astroglial Glu and Gln biosynthesis observed in AGC1 deficient probands. The lack of neuronal metabolic supply of NAA to oligodendrocytes and perhaps a direct effect on OPC proliferation/differentiation is responsible for the postnatal hypomyelination. In fact, a KD and KB (βOHB) administration to aralar-deficient humans and mice trigger resumed postnatal myelination, respectively. *aralar*-KO neurons have very low levels of Asp and NAA and this lack of neuronal NAA is recovered by βOHB administration to unmyelinated neurons from *aralar*-KO brain. This observation clearly supports that the lack of neuronal NAA supply is involved in this pathology. Finally, a direct effect of AGC1/Aralar deficiency on neuronal metabolism and excitability triggers the refractory epilepsy observed that is efficiently reduced with a KD in humans.

## Figures and Tables

**Table 1:** Molecular, biochemical, neuroimaging, and clinical findings in patients with inborn AGC1/Aralar deficiency or with other defects in the components of the malate-aspartate shuttle (MAS).

AGC1-Deficiency	Main Traits	Molecular	MRI	MRS	Biochemistry	Treatment
Wibom et al. (2009)	Delayed psychomotor development, seizures, hypotonia, spasticity and hyperreflexia	AGC1 (p.Gln590Arg)	Cerebral Hypomyelination, Reduced supratentorial cerebral volume, enlarged ventricles and subarachnoid space, reduced putamen	NAA ↓↓ Myoinositol ↑ Choline = Lactate = Lipids =	Lactate_plasma_ ↑↑ Lactate_CSF_ ↑ Aminoacids_plasma_ =	AED KD (*Dahlin* et al. *2015*)
Falk et al. (2014)	Profound developmental delay, congenital hypotonia, refractory epilepsy, multiple dysmorphic features.	AGC1 (p.Arg353Gln)	Global Hypomyelination, Enlarged subarachnoid spaces, ventricles and sulci Cerebral volume loss, Diffuse brain atrophy	NAA ↓↓ Choline ↑ Myoinositol ↑ Lactate (parenchyma and CSF) ↑	Lactate_plasma_ = Lactate_CSF_ = Aminoacids_plasma_ = Aminoacids_CSF_ =	AED
Parnes et al. (2015)	Delayed psychomotor development, epilepsy, hypotonia, spasticity and hyperreflexia, non-verbal	AGC1 (p.K100fs) (p.I72T)	Hypomyelination, brain atrophy	NAA ↓↓	NA	AED
Pronicka et al. (2016)	NA	AGC1 (p.Asn445Lys)	NA	NA	NA	AED
Pfeiffer et al. (2019)	Intractable epilepsy, psychomotor delay, cerebral atrophy, severe hypotonia	AGC1 (p.Thr444Ile)	Unaffected myelination Cortical brain atrophy Ventricles not mentioned	NAA ↓↓ Choline ↑ Myoinositol ↑ Lactate peak not resolved	Acylcarnitine = Lactate_plasma_ ↑ Lactate_UrineOrganicAcids_ ↑ Aminoacids_plasma_ =	KD
Kavanaugh et al. (2019)	Global developmental delay, optic neuropathy and visual impairment, spasticity and cerebral palsy, epilepsy without status epilepticus, non-verbal	AGC1 (p.A432V) (c.1447-2_1447-1delAG)	Diffuse white matter volume loss Increased ventricular volume and thinned corpus callosum Enlarged subarachnoid spaces and sulci	NAA ↓↓ Lactate ↑ (Hypothesized to be a seizure sequel)	NA	AED
Nashabat et al. (2019)	Refractory epilepsy, optic neuropathy and visual impairment, no hypotonia no microcephaly	AGC1 (p.Thr462Met)	Unremarkable	NA	NA	AED
Saleh et al. (2020)	Global developmental delay, epilepsy, no speech, hypertonia	AGC1 (p.Arg134 *)	Thin Corpus Callosum Diffuse brain atrophy Enlarged ventricles	NA	NA	NA
Kose et al. (2021)	NA	AGC1 (p.Arg42Pro)	NA	NA	NA	NA
**Other MAS Defects**	**Main Tracts**	**Molecular**	**MRI**	**MRS**	**Biochemistry**	**Treatment**
Broeks MH et al. (2019)	Global developmental delay, Infantile Epileptic Encephalopathy, progressive microcephaly, dysmorphic facies, axial hypotonia/hypertonia of extremities	MDH1 (p.Ala138Val)	Partial agenesis of corpus callosum, enlarged ventricles, mild hypoplasia of inferior vermis and pons	NA	Lactate_plasma_ = Aminoacids_plasma_ = Acylcarnitine = Organic Acids = Dried-Blood Spot: Glutamate ↑ G-3-P ↑	AED
Ait-El-MKadem S et al. (2017)	Generalized hypotonia, psychomotor delay and refractory epilepsy, muscle atrophy and dyskinesia	MDH2 (p.Pro133Leu) (p.Pro207Leu) (p.Gly199Alafs*10) (p.Gly37Arg)	Global brain atrophy, Corpus callosum atrophy, delayed myelination; cortical, frontal and parietal atrophy	Lactate ↑	Lactate_plasma_ ↑ Lactate_CSF_ ↑	AED, KD
Karnebeek et al. (2019)	Progressive microcephaly, hypotonia, myoclonic epilepsy, profound intellectual disability, spasticity, frequent infections, non-verbal	GOT2 (p.Leu209del) (p.Arg337Gly) (p.Arg262Gly) (p.Gly33Val)	Mild cerebral atrophy, thinned corpus callosum, hypoplastic vermis (only in 1 case: multicistic encephalomalacia and asymmetric dilated lateral ventricles	NA	Aminoacids_plasma_ = Lactate_plasma_ ↑ Ammonia_plasma_ ↑ Organic Acids and Acylcarnitine =	Pyridoxine and serine (2 out of 4 patients)

AED, antiepileptic drug; AGC1, aspartate-glutamate carrier 1; CSF, cerebrospinal fluid; GOT2, mitochondrial Glutamate oxaloacetate transaminase; KD, ketogenic diet; MDH1, cytosolic malate dehydrogenase; MDH2, mitochondrial malate dehydrogenase; NA, not available; NAA, *N*-acetylaspartate; *, stop.
